# Supplementary material for: Learning curve and surgical outcome of robotic assisted colorectal surgery with ERAS program
Source: Sci Rep. 2022 Nov 29;12:20566. doi: 10.1038/s41598-022-24665-w (PMC9709162; doi:10.1038/s41598-022-24665-w)
Supplement: Supplementary file 2 — Supplementary Information 2. [file 41598_2022_24665_MOESM2_ESM.zip › RAL Raw data-Ñ[▒K/130/HS_ñw╝╨░O▒Kñσ.pdf]

正式摘要(電子簽章：000000231362823)

W95 / W95 ☐英文標題(注意:無英文姓名-請補病人基本檔!) ☐【讀取】憑證

臺 中 榮 民 總 醫 院

出院病歷摘要

轉入號： 轉出號：

[redacted]

(8)流水編號	
(9)入院日期	110/11/30 (2021)
(10)轉科日期	
(11)出院日期	110/12/06 (2021) 住院天數計 6 日

診斷

- 入院 :1. S-colon cancer
  2. HTN under medication control
  3. R/I CKD, since 2019, medicine related(statin?)
- 出院 :1. S-colon cancer s/p Robotic assist AR on 2021/12/02
  2. HTN under medication control
  3. R/I CKD, since 2019, medicine related(statin?)

主訴

- Bloody tinged stool noticed for 3 weeks

病史

- This is a78 years old male patient with history of

  1. HTN under medication control

  2. R/I CKD, since 2019, medicine related(statin?)

  According to the patient, he had intermittent constipation sicne 2021/09 and blood tinged stool was noticed for 3 weeks. He visit LMD where UGI&LGI scopy were done, showed S-colon cancer with partial obstruction. Thus, he visited our CRS OPD for second opinion. Under the impression of S-colon cancer, this time he was admitted to our ward for surgical intervention.

體檢發現

-
  - General appearance :
    - easy looking , conscious clear , without cardiopulmonary distress
    - Vital signs自動帶入date 體溫(BT) 脈搏(P) 呼吸(R) 血壓(BP) 身高(BH) 體重(BW) BMI
20211130 36℃ 77次/分 18次/分 141/75mmHg 170.2cm 73.7kg 25.4
    - Vital signs: B.P. 141/75 mmHg , P.R. 77 /min , R.R. 18 /min , B.T. 36 ℃
  - HEENT :
    - Eyes: no anemic conjunctiva, no icteric sclera, normal EOM
    - ENT: no otorrhea, no rhinorrhea, no erythematous mucosa, no injected tonsil, no deformity
  - Neck: supple, freely movable, trachea midline,no deformity, no engorged jugular vein , no palpable thyroid or lymph node enlargement
  - Chest:
    - Inspection: Grossly normal, symmetric and fully expansion, no accessory muscle used, no operation scar, no spider angiomata, no gynecomastia
    - Palpation: no palpable mass or nodule, no subcutaneous emphysema, no tenderness
    - Ausculation: clear breathing sound
  - Heart:
    - Palpation: no thrill, no heave
    - Percussion: normal heart size
    - Ausculation: regular heart beat, normal S1, S2, no gallop, no murmur
  - Extremities: No deformity, full ranged of motion without limitation, no clubbing finger or nail cyanosis, no pitting edema, no ulcer or pigmentation, no obvious skin lesion, no varicose vein, no bone, joint or muscle tenderness

  CRS CONDITION

  - Karnofsky performance status: (100) Normal, no complaints, no evidence of disease
  - PE of Abdomen:
  - CEA: . ng/ml
  - Imaging Studies:
  - Colonoscope report: .
  - Figures:
    1. colon & rectum

手術日期及方法

- 手術日期及方法20211202 1. Robotic (XI) assisted anterior resection
  2. Intraoperative colonoscopy : (-).
  3. High ligation (+) : At IMA root. Left colic artery and IMV were also ligated via Endoclips.
  4. Take down from the splenic flexure : (-)
  5. Robotic endoGIA (45-3.5) X2 + EEA (33mm) were used
  6. Proximal cut end: 10 cm, distal cut end: 5 cm
  7. No drainage tube was inserted
  8. ADHESION BARRIER FILM was placed below the central abdominal wound

住院治療經過

- After admission, pre-OP abdominal CT was done on 12/01. He received Robotic assist Anterior Resection o n2021/12/02. He start try Bland I diet since 12/04. and had better appetite since 12/05. Under relative stable condition, he was discharged on 12/06, OPD f/u.

合併症

一般檢查

- 累積報告 :

DATE	TIME	ALB	TP	BIL.T	BIL.D	ALKP	AST	ALT	LDH	AMY	LIPASE	NH3	Ferritin	UA
1101130	1129	4.3		1.4	0.4	56	18	14	146					
1101202	0704		6.4											8.8
1101202	1848	3.3		1.1				10						
1101206	0911	3.0		1.18	0.39	41	13							

DATE	TIME	WBC	RBC	HGB	HCT	MCV	PLT	BLST	NEUT	BAND	SEG	LYM	MON	EOS	BAS
1101130	1129	7480	3.31	10.9	33.2	100.3	207		66.1			25.0	5.7	2.9	0.3
1101202	1844	9550		9.2	27.5		169		87.5			10.5	1.9	0.1	0.0
1101206	0932	6950		8.6	25.8		215		70.2			17.4	6.5	5.6	0.3

DATE	TIME	NA	K	CL	BUN	CREAT	eGFR	CA	Mg	P	CRP	ESR	PCT	D-Dimer	LACTATE
1101130	1129	139	4.6		26	1.67		9.0							
1101202	0704										0.578				
1101202	1848	139	3.9	106	17	1.32	55.75	8.0							
1101206	0911	141	2.8		14	1.61	44.33								

DATE	TIME	GLUCOSE	HbA1C	CHOL	TG	HDL	LDL	KETONE	CK	CKMB	TRO-T	PT-P	APTT-P	NT-pro BNP
1101130	1129	95										10.6	30.4	
1101202	0704			176	155	27	115							

- 放射線報告 :檢驗項目 **:CT ABDOMEN-SURVEY** 報告日期 **:1101205**

主　訴：
Result:Y
Finding:
Imaging Report Form for Colorectal Carcinoma (AJCC 8th)
1. Imaging Date: 20211201
Imaging Modality, Imaging by
CT scan
Imaging Protocol: CT of abdomen with IV and oral contrast enhancement:
2. Tumor location / Size
Location:
Sigmoid
Size:
Non-measurable
3. Tumor invasion
Tumor within colonic or rectal wall
4. Regional nodal metastasis
No
5. Distant metastasis (In this study)
No or Equivocal
6. Other findings: > A case of sigmoid colon cancer for staging
> Presence of asymmetric wall thickening of sigmoid colon(Ser:201, img:57) maybe compatible with Sigmoid colon cancer
> Some low attenuation lesions are noted over bilateral lobes of liver, favor hepatic cysts
> Presence of gallstone about 9mm in size
> pancreas, spleen, bilateral adrenal glands, bilateral kidneys, and bilateral lower lung fields shows no obvious abnormal
> No obvious enlarged lymph nodes are noted over retroperitonium, masentary, and bilateral inginal fossa
> Narrowing of disc space over L4-5
Impression:
1. Presence of asymmetric wall thickening of sigmoid colon(Ser:201, img:57) maybe compatible with Sigmoid colon cancer
2. For other details, please see the above descriptions.

特殊檢查

病理報告

其它

出院時情況

- 3.改門診治療

出院指示

-

藥名	劑量	劑量單位	用法	頻次	數量	數量單位	現況	天數	單位
Ultracet tab	1	TAB	PO	BIDPRN	18	TAB	TH	9	天
Norvasc tab 5mg	1	TAB	PO	QD	9	TAB	TH	9	天
$Biofermin tab	2	TAB	PO	TID	54	TAB	TH	9	天
Econ tab 40mg	1	TAB	PO	QID	36	TAB	TH	9	天

出院計劃

- 門診日期
  - date on 2021/12/15

主治醫師_2062F 蔣鋒帆_______________ / 住院醫師_2339C 蔡濬樟_______________

正式摘要(電子簽章：000000231362823)

W95 / W95

doctorRank:B
